# Polymer Entanglement-Induced Hydrogel Adhesion

**DOI:** 10.3390/gels10120822

**Published:** 2024-12-13

**Authors:** Kai Hu, Qingyun Li, Xiaofan Ji

**Affiliations:** 1 College of Chemistry and Chemical Engineering, Xi’an University of Science and Technology, Xi’an 710054, China; kaihu@stu.xust.edu.cn; 2 Key Laboratory of Material Chemistry for Energy Conversion and Storage, Ministry of Education, Hubei Key Laboratory of Material Chemistry and Service Failure, Hubei Engineering Research Center for Biomaterials and Medical Protective Materials, School of Chemistry and Chemical Engineering, Huazhong University of Science and Technology, Wuhan 430074, China; liqingyun@hust.edu.cn

**Keywords:** adhesion, hydrogel, polymer, supramolecular polymers

## Abstract

Hydrogels are widely used in the field of adhesive materials. However, hydrogel adhesion has previously required the covalent graft of supramolecular groups on polymeric chains. In contrast to that, here, a hydrogel adhesion induced by covalent polymer entanglement between two hydrogel networks was reported. Hydrogels **G1** and **G2** contain the monomers **M1**, with diazonium groups, and **M2**, with sulfonate groups, respectively. When the two hydrogels come into contact, the monomers diffuse into each other’s networks and assemble into supramolecular polymers (**SPs**) based on electrostatic interactions, threading the two hydrogel networks. Subsequently, **SPs** convert into covalent polymers (**CPs**) under UV light stimulation due to the reaction between the diazonium groups and sulfonate groups, leading to the entanglement of the two hydrogel networks and the production of an adhesive effect. This finding provides a novel strategy for hydrogel adhesion.

## 1. Introduction

The adhesion phenomenon widely occurs in nature; it is the outcome of interatomic and intermolecular interactions holding different surfaces together [1,2]. Among these adhesion phenomena, soft–soft adhesion commonly exists in our bodies, occurring between soft tissues and other kinds of tissues or organs. In addition, the binding of cells of various kinds is also referred to as soft–soft adhesion [3,4]. In regard to mimicking the adhesion behavior of soft tissues, hydrogel, as a specific kind of soft material, has gained people’s attention [5]. Benefitting from a hydrophilic polymer backbone, hydrogel holds a lot of water and performs macroscopic solid-like rheological behavior [6,7]. Hydrogels possess a rich array of properties, including a self-healing ability, a conductive ability, moldability, adjustable mechanical properties, a shape-memory ability, and a high water content, which have led to their extensive research in various fields, such as biomedicine, electronic sensors, conductive devices, soft robotics, and more [8]. In addition, hydrogels possessing tunable chemical structures, mechanic properties and biocompatibility are also regarded as exceptional adhesion materials [9,10]. Benefitting from these advantages, the adhesion of hydrogels finds important applications in different fields, such as medicine and biological materials [11,12].

Normally, hydrogel adhesion is achieved by noncovalent interactions [13,14,15,16]. Therein, noncovalent interactions embody hydrogen-bonding interactions, metal coordination, electrostatic interactions, and host–guest interactions [17,18]. Noncovalent interactions are reversible and adaptive, showing sensitivity to environments, which allows for facilitating the construction of hydrogel adhesion [19,20,21,22]. The Harada group has made many pioneering contributions in terms of utilizing the supramolecular recognition interactions between cyclodextrin macrocycles and different guest molecules, such as *β*-cyclodextrin/ferrocene, *β*-cyclodextrin/pyrene, *β*-cyclodextrin/dansyl, and *β*-cyclodextrin /benzyl to achieve hydrogel adhesion and regulate its adhesiveness [23]. Furthermore, Shi, Chen and coworkers reported a kind of hydrogel adhesion based on the host–guest interactions between *β*-cyclodextrin and azobenzene [24]. Huang, Sessler, and their colleagues constructed fluorescent hydrogel assemblies utilizing the host–guest interactions between tetracationic imidazolium macrocycle and sulfonates, which can be used as information storage materials [25]. Stemming from metal-coordination interactions, another hydrogel adhesion example was reported by He, Zhang, and coworkers, who reported that self-adhesive poly (acrylic acid)–Al^3+^ hydrogel served as a wearable strain hydrogel sensor [26]. Also, hydrogel adhesion was enabled by electrostatic interactions for which Sun, Wang, and coworkers fabricated strong electrolyte polyampholyte hydrogels via introducing a weak anionic monomer [27]. By means of different kinds of noncovalent interactions, the desired hydrogel adhesion is surely feasible [28,29,30]. However, current approaches for realizing hydrogel adhesion require the covalent graft of supramolecular groups on polymeric chains, entailing a complex synthesis process.

**Scheme 1:**
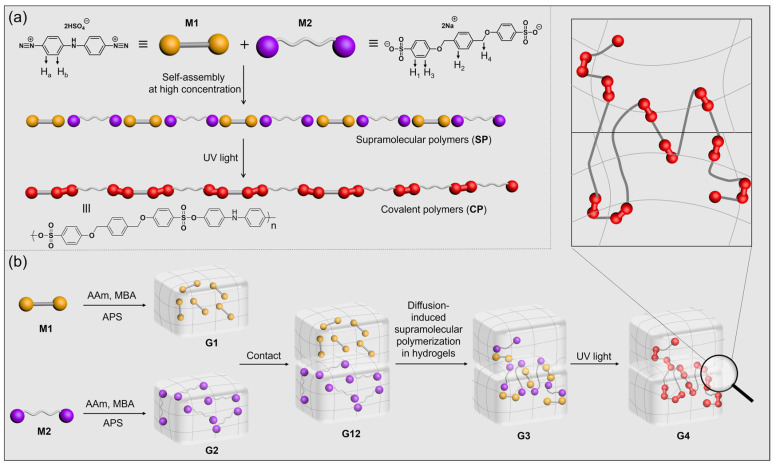
(**a**) Cartoon representation of the formation of supramolecular polymers (**SPs**) assembled by monomers **M1** and **M2** as well as the formation of covalent polymers (**CPs**); (**b**) schematic illustration of the preparation process of hydrogel **G4** and the adhesion behaviors achieved by the entanglement of polymers inside the hydrogel networks. The orange balls represent the benzenediazonium groups, and the purple balls represent the benzenesulfonic acid groups.

In contrast to previous methods, we put forward an approach that involves forming covalent polymers inside hydrogels to entangle different hydrogel networks, further achieving hydrogel adhesion. Monomer **M1**-containing diazonium groups and **M2**-bearing sulfonate groups were synthesized (Scheme 1a). Based on the electrostatic interaction between the diazonium groups and sulfonate groups, self-assembled behaviors between **M1** and **M2** occurred in water to form supramolecular polymers (**SPs**). Due to the reaction ability of supramolecular recognition groups to react under UV light stimulation to form covalent compounds [31], **SPs** could be transformed into covalent polymers (**CPs**) upon UV irradiation. Then, two kinds of hydrogels **G1** and **G2**, can be fabricated from acrylamide (AAm), *N*,*N′*-methylenebis (acrylamide) (MBA) and ammonium peroxidisulfate (APS) by means of radical polymerization [32]. Furthermore, **M1** and **M2** were incorporated into these hydrogels through physical blending (Scheme 1b). **G1** and **G2** were placed together for contacting each other, and, after 13 days passed, two hydrogels were fully contacted with the adequate diffusion of **M1** and **M2**. Therefore, as a result of **M1** and **M2** undergoing supramolecular polymerization, **SPs** were formed inside hydrogel **G3** networks, threading through different hydrogel networks. **SPs** could be transformed into **CPs** upon UV light irradiation. Therefore, the **CPs** in **G4** caused different hydrogel networks to be entangled, leading to the adhesion of two hydrogels. This method was used as a good candidate to achieve hydrogel adhesion, leading to the development of polymer science, hydrogel materials, and stimulation-responsive materials.

## 2. Results and Discussion

**Scheme 2:**
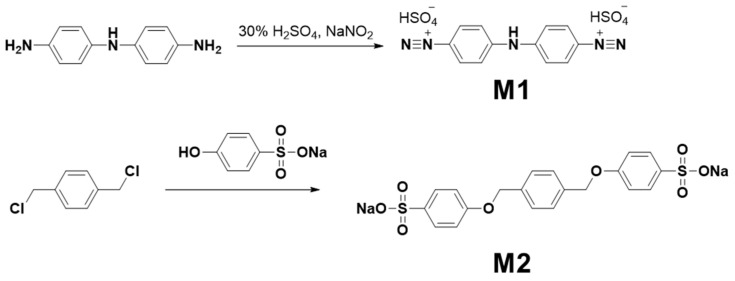
Synthesis route of monomers **M1** and **M2**.

Monomers **M1** and **M2** were synthesized according to Scheme 2. Moreover, the chemical structures of monomers have been confirmed by ^1^H NMR spectroscopy and ^13^C NMR spectroscopy (Figures S1–S3). **M1** (83.4 mg, 10 mM) and **M2** (98.8 mg, 10 mM) were dissolved in deionized water to prepare the stock solution. Furthermore, the standard solutions of **M1** and **M2** were prepared by diluting the stock solution via adding the deionized water. The conventional preparation method for hydrogels (Table S1) was as follows: AAm, MBA, and APS, were dissolved in deionized water and allowed to form the hydrogel. **M1** and **M2** were introduced into hydrogels through physical blending, resulting in hydrogels **G1** and **G2**, respectively. These hydrogels were then brought into contact for subsequent testing. Potential changes in the monomer’s structures during the UV light stimulation process were recorded using ^1^H NMR spectra, FT-IR spectra, UV-vis spectra, and gel permeation chromatography (GPC) spectra. The experimental details of the preparation and tensile testing of hydrogels are written in the Materials and Methods section.

### 2.1. The ^1^H NMR Tests and Viscosity Tests of the Mixture Solution of **M1** and **M2**

**Figure 1:**
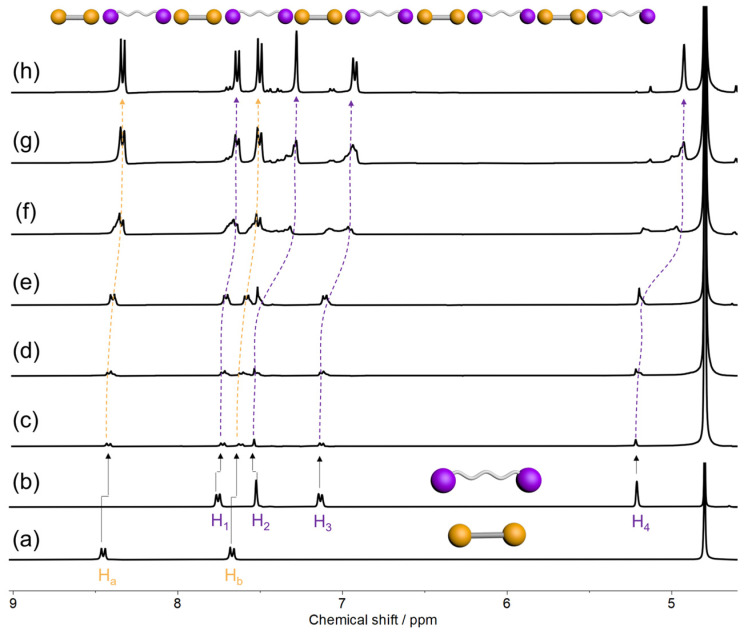
Partial ^1^H NMR spectra (400 MHz, D_2_O, 298 K) of (a) **M1** (10.0 mM), (b) **M2** (10.0 mM) and the mixtures of **M1** and **M2** at different concentrations of (c) 0.500 mM, (d) 1.00 mM, (e) 2.00 mM, (f) 5.00 mM, (g) 8.00 mM and (h) 10.0 mM. The orange balls represent the benzenediazonium groups, and the purple balls represent the benzenesulfonic acid groups.

In order to study the self-assembly behavior of **M1** and **M2** in water, the mixtures of **M1** and **M2** at different concentrations (0.500 mM–10.0 mM) in D_2_O were tested by ^1^H NMR spectra. As shown in Figure 1a–c, after mixing equimolar **M1** and **M2**, the signal peak H_a_ of **M1** shifted from 8.46 ppm and 8.44 ppm to 8.43 ppm and 8.40 ppm, respectively, and the H_b_ signal peak H_b_ shifted from 7.68 ppm and 7.65 ppm to 7.63 ppm and 7.60 ppm, respectively. Meanwhile, in regard to **M2**, H_1_ signal peaks shifted from 7.77 ppm and 7.75 ppm to 7.74 ppm and 7.72 ppm, respectively; H_2_ shifted from 7.52 ppm to 7.54 ppm; the signal peaks H_3_ shifted from 7.14 ppm and 7.12 ppm to 7.13 ppm and 7.11 ppm, respectively; and the methylene signal peak (H_4_) shifted from 5.21 ppm to 5.22 ppm. With the concentration of the mixture increasing from 0.500 mM to 10.0 mM (Figure 1c–h), H_a_ signal peaks at 8.43 ppm and 8.40 ppm shifted to 8.34 ppm and 8.32 ppm, respectively, exhibiting a phenomenon of shifting towards the upfield. Meanwhile, the signal of H_b_ shifted upfield, slightly changing from 7.63 ppm and 7.60 ppm to 7.51 ppm and 7.49 ppm, respectively. Similarly, from the spectra, upfield shifts of H_1_ signals ranging from 7.73 ppm and 7.71 ppm to 7.65 ppm and 7.62 ppm, respectively, were detected. In addition, the proton peaks (H_2_, H_3_) of the benzene group shifted to 7.28 ppm, 6.94 ppm, and 6.92 ppm, respectively, and the methylene protons (H_4_) in monomer **M2** underwent significant shifts towards the upfield, shifting from 5.22 ppm to 4.93 ppm. In addition, the above-mentioned peak shapes of signals became broader at 10.0 mM. The proton peaks changes could be attributed to the electrostatic interactions between diazonium groups and sulfonate groups [33]. Regarding the changes in broader peak shapes at high concentrations, it was revealed that only polymeric assemblies were formed [33].

**Figure 2:**
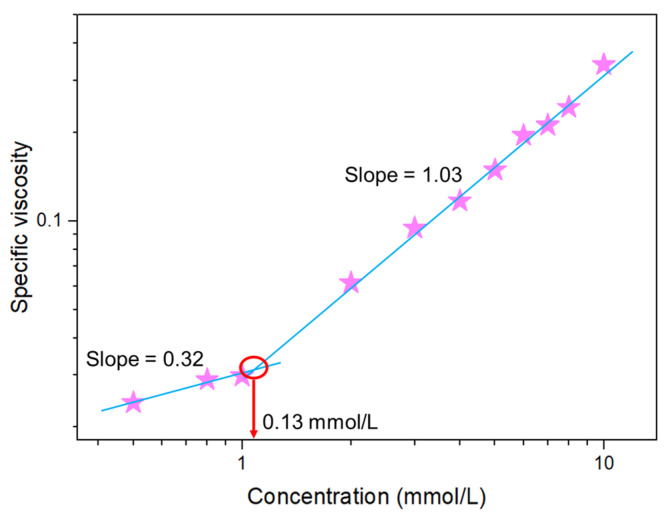
Plot of the specific viscosity of equimolar mixtures of **M1** and **M2** in H_2_O versus **M1** concentration at 298 K. The pink star represents the specific viscosity corresponding to a certain monomer concentration. The red circle represents the critical polymerization concentration (CPC).

For further studying the self-assembly behavior of **M1** and **M2** in an aqueous solution, a viscosity test was conducted. As the concentration of **M1** increased from 0.500 mM to 1.00 mM, the specific viscosity of the mixture solution gradually rose with the slope of 0.32 (Figure 2). With the concentration of **M1** exceeding over 2.00 mM, the specific viscosity of the mixture solution increased significantly with a slope of 1.03. The concentration of monomer corresponding to the intersection point of the two fitted lines (at 0.13 mM) was defined as the critical polymerization concentration (CPC) [34]. The slope within the low-concentration range reflected the characteristics of cyclic species or oligomers expected in dilute solutions. In contrast, the sharp increase in specific viscosity above the CPC was attributed to the formation of linear **SPs** [34]. Therefore, these results furtherly confirmed that **SPs** could be assembled by monomers at high concentrations while relatively smaller oligomers could form at lower monomer concentrations.

### 2.2. The ^1^H NMR Tests, FT-IR Tests, TGA Tests, and GPC Tests of the Mixture Solution of **M1** and **M2** After UV Light

**Figure 3:**
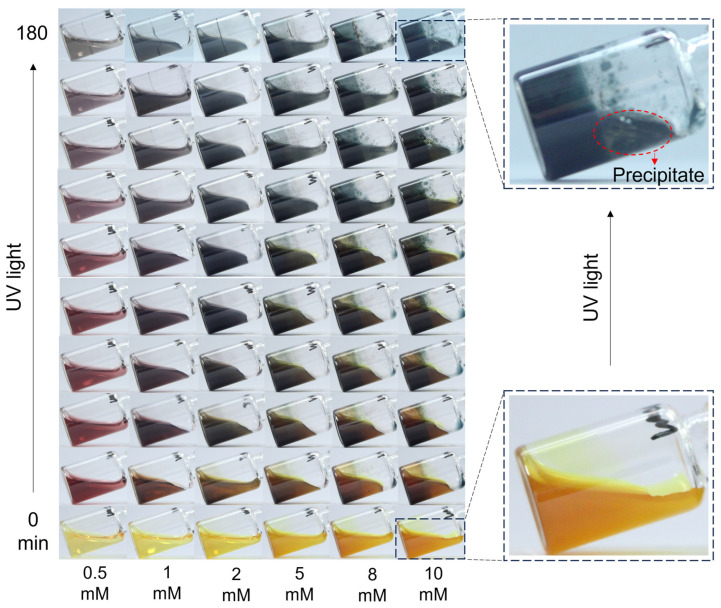
Photographs of the mixture solutions of **M1** and **M2** at different times (0–180 min) under UV light.

Based on the formation of **SPs**, we further explore the formation of **CPs**. According to the reported literature, diazonium groups and sulfonate groups could react under UV light to form covalent compounds. If such reactions can proceed, the newly formed compounds will not contain water-soluble groups and will precipitate out from water. Therefore, the observation of precipitation in the reaction solution can serve as evidence for the occurrence of such reactions. As shown in Figure 3, the color of initial mixed solutions gradually changed from a pale yellow to an orange color after the increase in the monomer concentration. After exposure to UV light, the solution containing 0.500 mM monomers underwent a significant color change from pale yellow to burgundy, and, as the exposure time increased to 180 min, the solution color ultimately turned gray. As the concentration of the solution further increased, the color of the solution after the same exposure time darkened further and a black precipitate gradually formed on the walls of the container. As mentioned above, the change in color and the formation of the precipitate in the mixed solution indicated that **M1** could react with **M2** under UV light to produce water-insoluble compounds [31,35]. This was potentially a reaction between diazonium groups and sulfonate groups.

**Figure 4:**
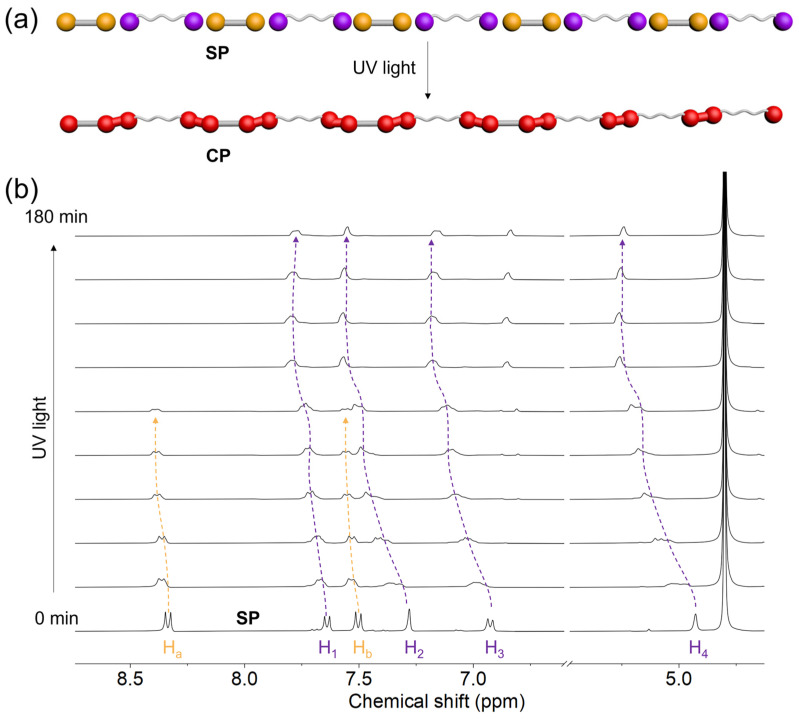
(**a**) Representation of the formation of **CPs** transformed from **SPs** under UV light; (**b**) partial ^1^H NMR spectra (400 MHz, D_2_O, 298 K) of **SPs** assembled by **M1** (10.0 mM) and **M2** (10.0 mM) under UV light at different times (0–180 min).

To further investigate the reasons for the aforementioned solution changes, a mixture solution containing 10.0 mM monomers was subjected to UV light while simultaneously recording ^1^H NMR spectra (Figure 4a). After UV light stimulation, the proton peaks of monomer **M1** (H_a_–H_b_) in the solution shifted from 8.34 ppm, 8.32, ppm, 7.51 ppm, and 7.49 ppm to 8.40 ppm, 8.38 ppm, 7.57 ppm, and 7.55 ppm, and the signals of its proton peaks disappeared after 30 min. Meanwhile, the signal peaks of monomer **M2** also underwent an initial shift towards the downfield, moving from 7.65 ppm, 7.27 ppm, 6.93 ppm, and 4.97 ppm to 7.75 ppm, 7.73 ppm, 7.52 ppm, 7.13 ppm, and 5.20 ppm, then shifting to 7.78 ppm, 7.54 ppm, 7.16 ppm, and 5.24 ppm, respectively. Within 30 min of UV light irradiation, significant shifts were observed in the proton peak signals of the benzene group in **M1** and **M2**, which could be inferred to indicate structural changes in the diazonium groups and sulfonate groups, suggesting that a chemical reaction occurred between these two groups [33]. Based on these data, further speculation could be made that **SPs** transformed into **CPs** under UV light irradiation [35]. In addition, after 30 min of UV irradiation, the disappearance of the signal peak of **M1** could be attributed to a partial reaction between **M1** and **M2** as well as the decomposition of diazonium groups in some **M1** under light, leading to its precipitation from the water. According to the previous literature [36,37], the diazonium salt group may react with phenol derivatives or D_2_O to form ether under prolonged UV light exposure (Scheme S1). Therefore, the signal peak at 6.70 ppm could be attributed to the hydrogen protons on the benzene ring of the compound 4,4′-azanediyldiphenol. Therefore, the unreacted monomer **M2** and 4,4′-azanediyldiphenol remained in the solution after 180 min of UV light irradiation.

**Figure 5:**
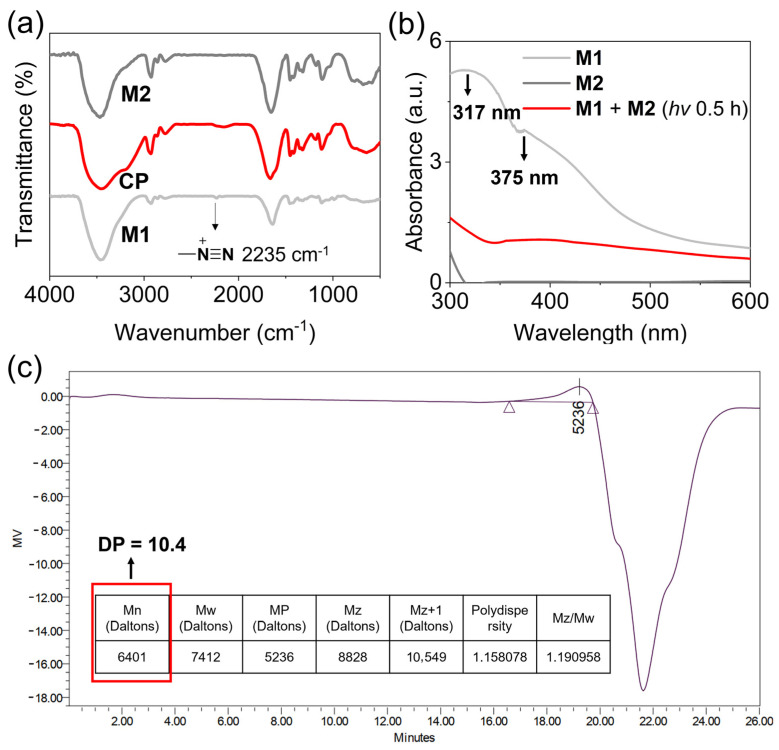
(**a**) FT-IR spectra of **M1**, **M2** and **CP**; (**b**) UV-vis spectra of **M1** (10.0 mM), **M2** (10.0 mM), and the mixture of **M1** and **M2** after being exposed to UV light for 0.5 h; (**c**) GPC curve of **CP**.

In addition to monitoring the structural changes in compounds in solution, the precipitates formed after photoreaction also require further investigation. FT-IR technology was employed to record the spectra of monomers and the precipitates. As shown in Figure 5a, the signal peak at 2235 cm^−1^ was attributed to the diazonium groups in **M1**. Notably, the diazonium signal peak was absent in the precipitates, further confirming the reactions between the diazonium groups and the sulfonate groups [38] as well as the formation of covalent polymers. As shown in Figure 5b, the **M1** solution exhibited two distinct signal peaks at 317 nm and 375 nm, recorded in the UV-vis spectra, attributed to the diazonium groups of **M1** [31]. Additionally, **M2** did not exhibit any peaks within this wavelength range. After the mixed solution was exposed to UV light for 0.5 h, these signal peaks corresponding to the diazonium groups disappeared and a significant decrease in absorbance intensity was observed. This evidence indicated the decomposition of the diazonium salt structure and the possible formation of new compounds. Subsequently, gel permeation chromatography (GPC) testing was conducted on the covalent polymers to investigate their molecular weights. As illustrated in Figure 5c, a broad peak appeared within the retention time of 18–20 min, with an integrated number-average molecular weight of 6401. Further calculation yields a degree of polymerization of 10.4. These mentioned data demonstrated that the **CPs** were formed through the reaction between **SPs**, specifically via the reaction between the diazonium groups and sulfonate groups.

### 2.3. The Tensile Tests of Hydrogels **G01’**, **G02’**, and **G4**

**Figure 6:**
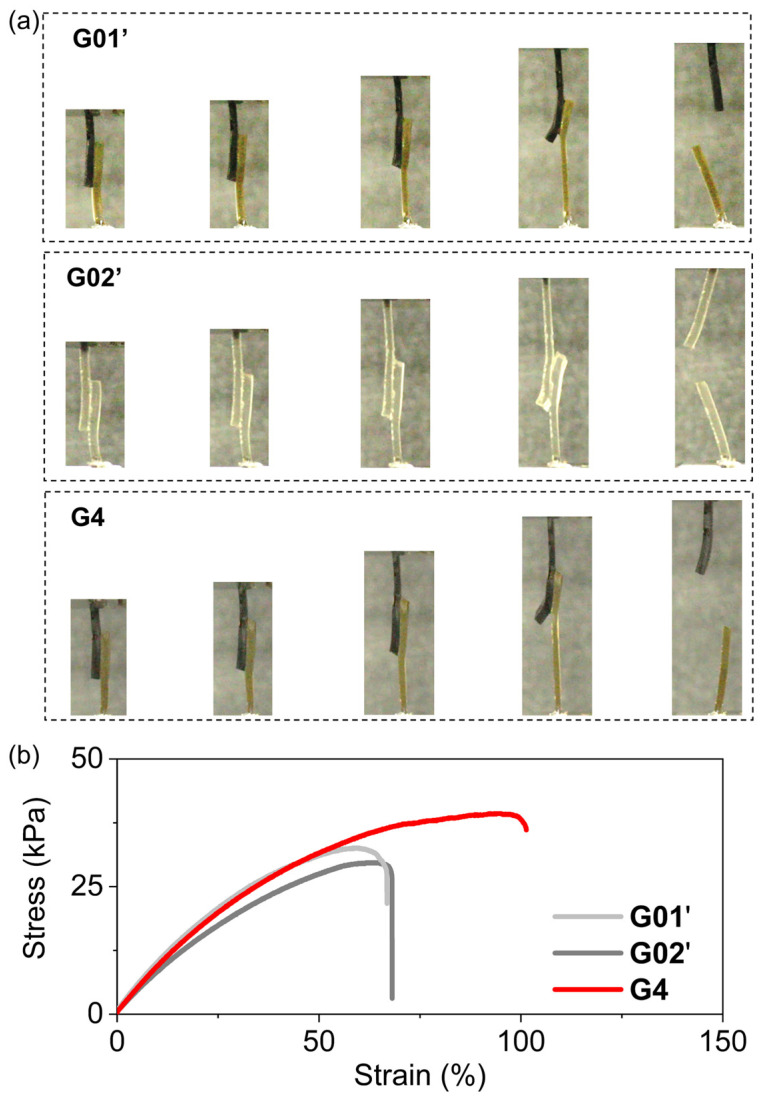
(**a**) Photographs of **G01’**, **G02’** and **G4** in the tensile process; (**b**) stress–strain curves of **G01’**, **G02’**, and **G4**.

With the aim to study hydrogel adhesion induced by polymer entanglement, **G1** and **G2** were contacted and subsequently treated with UV light for testing the adhesion behaviors. Hydrogels **G1** and **G2** were allowed to contact each other for 13 days before being subjected to UV irradiation for 30 min for tensile testing. Meanwhile, the control groups **G01’** and **G02’** were obtained by contacting the blank hydrogel **G0** with **G1** and **G2**, respectively, for 13 days, followed by UV light treatment. As shown in Figure 6a and Movie S1, at a speed of 5 mm min^−1^, after undergoing a tensile displacement, the hydrogels **G01’**, **G02’**, and **G4** all exhibited dissociation behavior (Figures S4 and S5). In the stress–strain curves (Figure 6b), the ultimate tensile stress and strain of **G4** were 38.7 kPa and 99.12%, respectively, which were significantly higher than those of the control groups (the ultimate tensile stress and strain of **G01’** were both 32.0 kPa and 62.86%, respectively; the ultimate tensile stress and strain of **G02’** were also both 28.9 kPa and 67.28%, respectively). This suggested that, during prolonged contact between the hydrogels, the two monomers diffused into the other hydrogel network and formed **SPs** through electrostatic interactions, entangling the two hydrogel networks at the interface [39,40]. Subsequently, under UV light treatment, the **SPs** converted into **CPs**, causing the entanglement of the two hydrogel networks to become even tighter [41,42] and resulting in stronger hydrogel adhesion. Compared to the state where monomers were uniformly distributed in solution, monomers tended to accumulate at the gel interface due to their diffusion behaviors [43]. This monomer aggregation phenomena favored the formation of longer supramolecular polymers at the interface [44], leading to the formation of longer covalent polymers and a good adhesion effect.

## 3. Conclusions

In summary, we reported a design for achieving hydrogel adhesion by means of forming covalent polymers inside hydrogels to entangle different hydrogel networks. At higher concentrations, **M1** could assemble with **M2** to form **SPs** based on electrostatic interactions. The formation of **SPs** was verified via conducting ^1^H NMR spectra and viscosity studies. Upon UV light stimulation, **SPs** transformed into **CPs**, which was confirmed through characterization by FT-IR spectroscopy and GPC. **M1** and **M2** were introduced into hydrogels, respectively, and these two hydrogels were placed into contact with each other. Upon the time passing, adequate diffusion of **M1** and **M2** proceeded, thereby forming **SPs** based on electrostatic interactions. The **SPs** were threaded throughout hydrogel networks. Subsequently, under UV light exposure, the **SPs** transformed into **CPs**, causing further entanglement between these two hydrogel networks. Then, tensile tests confirmed the hydrogel adhesion. This approach allowed for offering a new strategy for hydrogel adhesion, avoiding the complex covalent graft of supramolecular groups on polymeric chains. In addition, this new adhesion mechanism broadened the application scope of covalent polymers and promoted the development of supramolecular chemistry, polymer materials, photoresponsive materials, adhesion materials, and hydrogel materials.

## 4. Materials and Methods

All reagent materials mentioned in this article were purchased from commercial suppliers and were used directly without any additional treatment. 4,4′-iminobisbenzenamine, D_2_O, DMSO-*d*_6_, 4-hydroxybenzenesulfonate sodium and LiBr were purchased from Energy Chemical company (Wuhan, China). H_2_SO_4_, NaNO_2_, acetone and dichloromethane were obtained from SINOPHARM (Shanghai, China). 1,4-bis(chloromethyl)benzene, potassium carbonate, acrylamide and ammonium peroxydisulfate were purchased from Aladdin (Shanghai, China). *N,N′*-methylenebis(acrylamide) was obtained from Macklin (Shanghai, China). DMF (HPLC grade) was obtained from Thermo Fisher Scientific Inc. (Shanghai, China). The monomers **M1** and **M2** were synthesized according to procedures found in the literature [29,35]. ^1^H NMR spectra and ^13^C NMR spectra studies were performed using a Bruker Advance 400 MHz spectrometer (Billerica, MA, USA). Fourier transform infrared (FT-IR) spectra were recorded on a Bruker Equinox 55 (Billerica, MA, USA). Gel permeation chromatography (GPC) analyses were performed on a Waters 1515 instrument (Milford, MA, USA) equipped with a Waters 4.6 × 30 mm guard column and three Waters WAT054466, WAT044226, and WAT044223 columns (Polymer Laboratories: linear range of molecular weight = 500 − 4 × 10^6^), as well as a differential refractive index detector, using DMF (HPLC grade, containing 50 mmol/L LiBr) as the eluent at 35 °C with a flow rate of 1 mL min^−1^. An electronic universal testing machine (CMT4104, Shenzhen San Testing Machine Co., Shenzhen, China) was used to investigate the mechanical properties of hydrogels.

### 4.1. Synthesis and Characterization of **M1** and **M2**

In a 100 mL flask, 4,4′-iminobisbenzenamine (0.021 mol, 4.19 g) was mixed with crushed ice (30 g) and 30% H_2_SO_4_ (40 mL) (Scheme S1) [29,35]. Subsequently, a solution containing NaNO_2_ (0.050 mol, 3.45 g) in 10 mL water was added dropwise to the mixture. The mixed solution was then allowed to react in an ice-water bath for 2 h before being filtered to collect the filtrate. Concentrated H_2_SO_4_ was added to the filtrate to precipitate the diazonium salt, yielding monomer **M1**. ^1^H NMR (400 MHz, D_2_O) δ 8.45 (d, *J* = 9.2 Hz, 4H), 7.67 (d, *J* = 9.4 Hz, 4H).

In dry acetone (15 mL), 1,4-bis(chloromethyl)benzene (1.4 mmol, 243 mg) was mixed with 4-hydroxybenzenesulfonate sodium (3.5 mmol, 686 mg) and dried potassium carbonate (3.5 mmol, 483 mg), and the mixture was heated under N_2_ with reflux for 48 h [45]. After the reaction was completed, the mixture was allowed to cool and then evaporated to dryness under reduced pressure. The residue was extracted with dichloromethane. The aqueous layer was washed twice with dichloromethane and the organic layers were combined. The crude product was recrystallized from a mixture of dichloromethane and hexane, yielding monomer **M2**. ^1^H NMR (400 MHz, DMSO-*d_6_*) δ 7.51 (d, *J* = 8.5 Hz, 4H), 7.45 (s, 4H), 6.93 (d, *J* = 8.6 Hz, 4H), 5.11 (s, 4H). ^13^C NMR (100 MHz, D_2_O) δ 160.18, 136.33, 135.18, 128.38, 127.38, 115.10, 70.02.

### 4.2. Preparation of Hydrogels **G0**, **G1** and **G2**

Acrylamide (9.64 mmol, 0.684 g), *N,N′*-methylenebis(acrylamide) (5.96 × 10^−3^ mmol, 0.924 mg) and ammonium peroxydisulfate (19.7 × 10^−3^ mmol, 4.50 mg) were dissolved in 2.00 mL of deionized water to obtain a precursor solution. The precursor solution was poured into a glass mold with an array of cubic cavities (10 × 10 × 0.2 cm, length × width × height). The mold was then left at R.T. for 10 h to obtain the blank hydrogel **G0**. Following this preparation method of hydrogel **G0**, **M1** (10 mM) and **M2** (10 mM) were separately added to obtain hydrogels **G1** and **G2**, respectively.

### 4.3. Preparation of Hydrogels **G01’**, **G02’** and **G4**

The hydrogels **G0**, **G1**, and **G2** were each cut into rectangular strips (2 × 0.5 cm, length x width). Subsequently, strip **G0** was placed in contact with strips **G1** and **G2**, respectively, with a contact area of 0.5 × 0.5 cm. The samples were then stored in sealed bags in the dark for 13 days. After this period, the strips were taken out and exposed under a mercury lamp for 0.5 h. Following the UV light exposure, tensile tests were performed on the strips (**G01’** and **G02’**). The preparation of the strip **G4** followed the same preparation procedure.

## Data Availability

Data are contained within the article and Supplementary Materials. The original contributions presented in the study are included in the article and Supplementary Materials, further inquiries can be directed to the corresponding author.

## Supplementary Materials

The following supporting information can be downloaded at: https://www.mdpi.com/article/10.3390/gels10120822/s1. Figure S1: ^1^H NMR spectrum (400 MHz, D_2_O, 298 K) of **M1**; Figure S2: ^1^H NMR spectrum (400 MHz, DMSO-*d*_6_, 298 K) of **M2**; Figure S3: ^13^C NMR spectrum (100 MHz, D_2_O, 298 K) of **M2**; Figure S4: Stress-strain curves of **G3** and **G4**; Figure S5: The tensile curves of hydrogels (a) **G01**, (b) **G02**, and (c) **G4** as well as (d) summarized final fractured strain and final fractured stress of themselves. Movie S1: Tensile tests of **G01’**, **G02’** and **G4**. Table S1: Preparation of hydrogels; Scheme S1: Synthesis route of 4,4′-azanediyldiphenol-d.
