# Supplementary material for: Polymer Entanglement-Induced Hydrogel Adhesion
Source: Gels. 2024 Dec 13;10(12):822. doi: 10.3390/gels10120822 (PMC11675780; doi:10.3390/gels10120822)
Supplement: Supplementary file 1 [file gels-10-00822-s001.zip › gels-3305055-supplementary.pdf]

*Article*

# Polymer Entanglement-Induced Hydrogel Adhesion

**Kai Hu [1], Qingyun Li [2] and Xiaofan Ji [2,*]**

[1] College of Chemistry and Chemical Engineering, Xi'an University of Science and Technology, Xi'an 710054, China; kaihu@stu.xust.edu.cn
[2] Key Laboratory of Material Chemistry for Energy Conversion and Storage, Ministry of Education, Hubei Key Laboratory of Material Chemistry and Service Failure, Hubei Engineering Research Center for Biomaterials and Medical Protective Materials, School of Chemistry and Chemical Engineering, Huazhong University of Science and Technology, Wuhan 430074, China; liqingyun@hust.edu.cn
* Correspondence: xiaofanji@hust.edu.cn

## Supplementary Materials

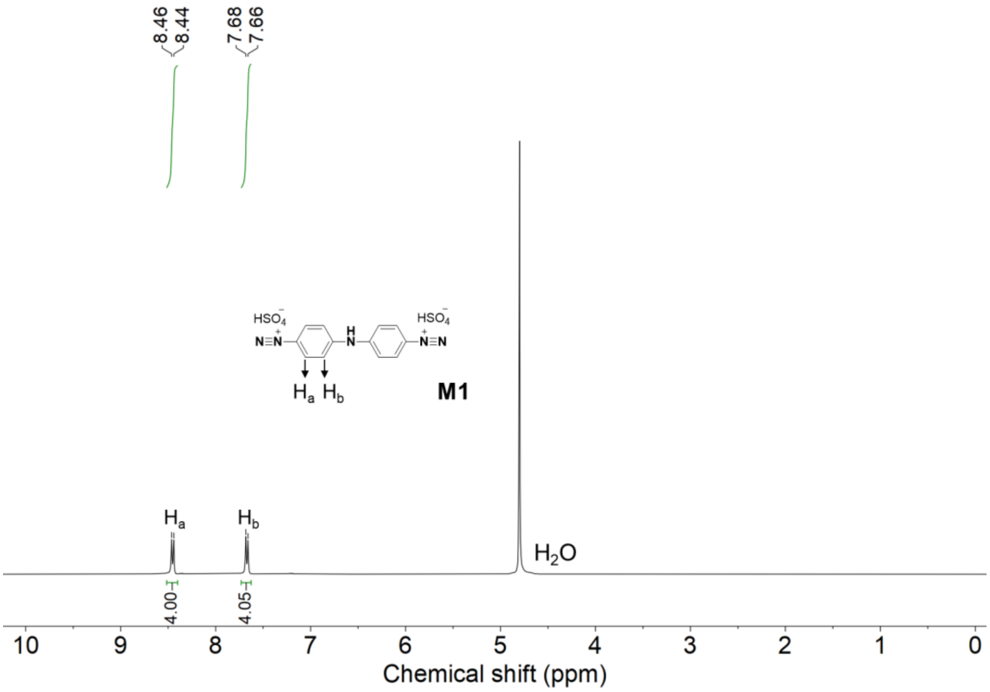


**Figure S1.** $^1$H NMR spectrum (400 MHz, $D_2O$, 298 K) of **M1**.

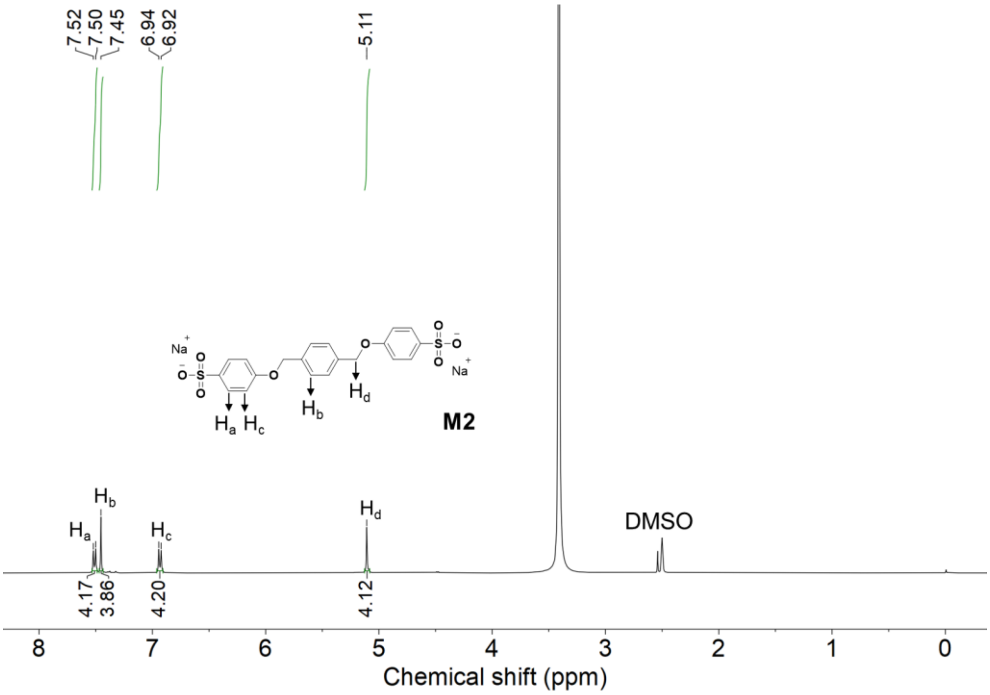


**Figure S2.** $^{1}$H NMR spectrum (400 MHz, DMSO-$d_6$, 298 K) of **M2**.

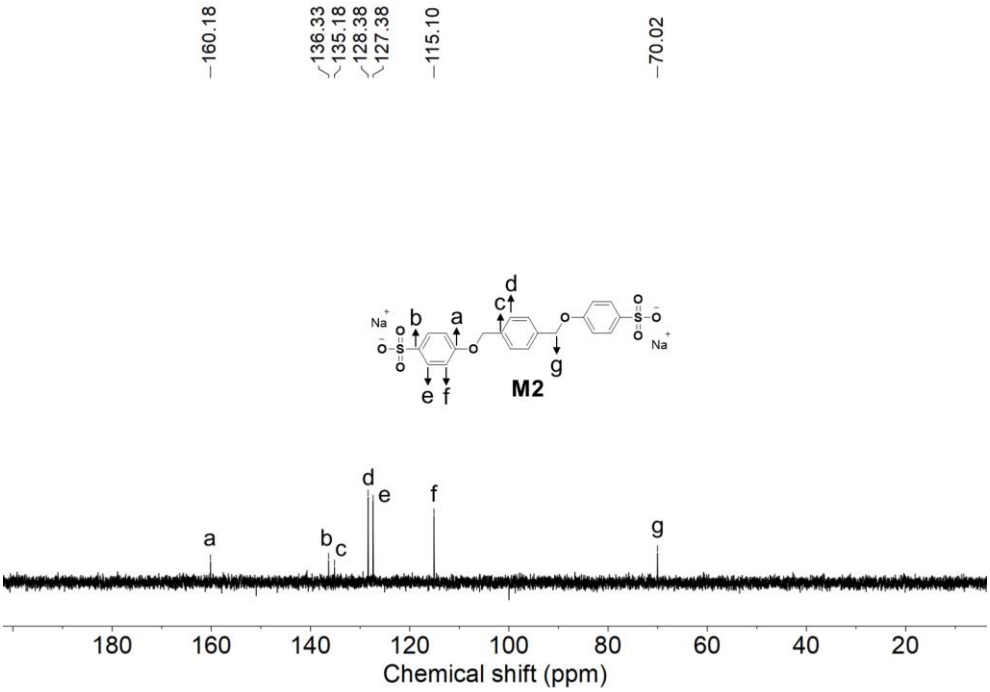


**Figure S3.** $^{13}$C NMR spectrum (100 MHz, $D_2O$, 298 K) of **M2**.

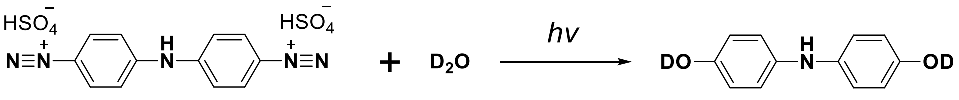


**Scheme S1.** Synthesis route of 4,4'-azanediyldiphenol-*d*.

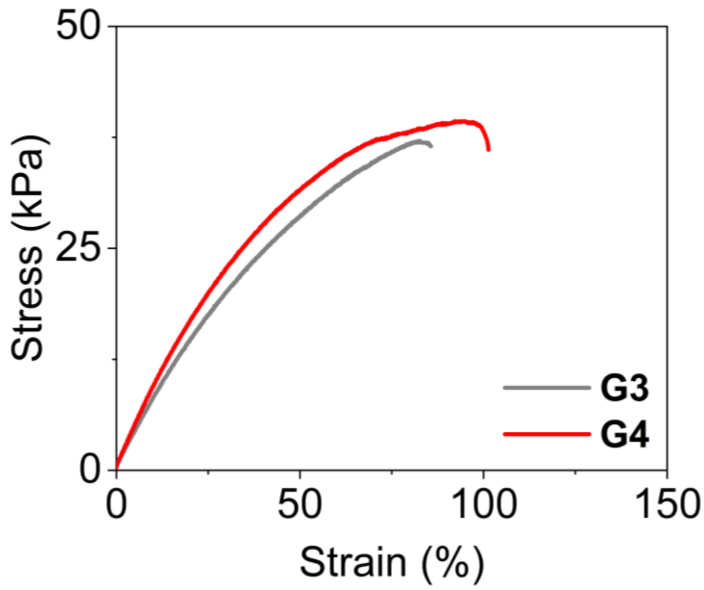


**Figure S4.** Stress-strain curves of **G3** and **G4**.

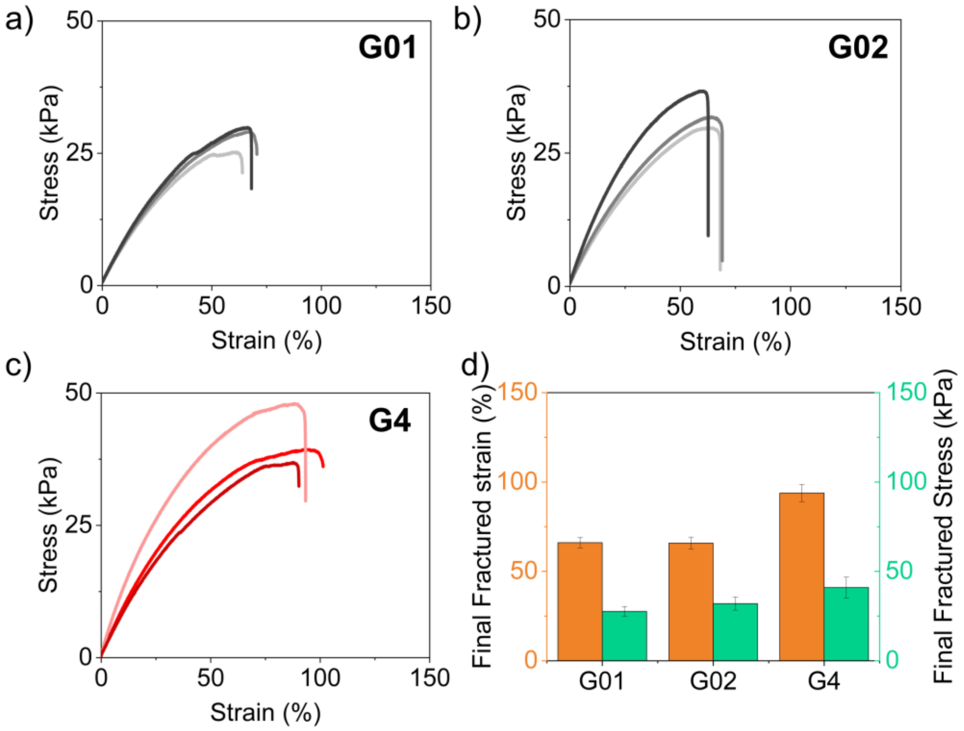


**Figure S5.** The tensile curves of hydrogels (a) **G01**, (b) **G02**, and (c) **G4** as well as (d) summarized final fractured strain and final fractured stress of themselves.

**Table S1.** Preparation of hydrogels.

| Compositions | Acrylamide | *N,N′*-methylenebis(acrylamide) | Ammonium peroxydisulfate | **M1** | **M2** | $H_2O$ |
|---|---|---|---|---|---|---|
| **G0** | 0.684 g | 0.924 mg | 4.50 mg | 0 | 0 | 2 mL |
| **G1** | 0.684 g | 0.924 mg | 4.50 mg | 8.34 mg | 0 | 2 mL |
| **G2** | 0.684 g | 0.924 mg | 4.50 mg | 0 | 9.88 mg | 2 mL |
